# Adolescent Substance Use Screening in the Emergency Department

**DOI:** 10.1016/j.acepjo.2026.100447

**Published:** 2026-06-22

**Authors:** Stephen Sandelich, Siraj Amanullah, Isabella Barata, Kathleen Berg, Kathleen Brown, Tabitha Cheng, Corrie Chimptazi, Ilene Claudius, Ann M. Dietrich, Sophia Lin, Tim Ruttan, Michael Stoner, Carmen Sulton, Muhammad Waseem

**Affiliations:** 1 Department of Emergency Medicine and Pediatrics, Penn State College of Medicine, Hershey, Pennsylvania, USA; 2 Department of Emergency Medicine, Pediatrics, and Health Services, Policy and Practice, Alpert Medical School of Brown University and Brown School of Public Health, Hasbro Children's Hospital/Rhode Island Hospital, Providence, Rhode Island, USA; 3 Department of Pediatrics and Emergency Medicine, Zucker School of Medicine at Hofstra/Northwell, Uniondale, New York, USA; 4 Department of Pediatrics, University of Texas at Austin-- Dell Medical School, Austin, Texas, USA; 5 Department of Pediatrics and Emergency Medicine, the George Washington University (GWU) School of Medicine, Washington, District of Columbia, USA; 6 Department of Emergency Medicine, David Geffen School of Medicine at University of California, Los Angeles (UCLA), Harbor-UCLA Medical Center, Department of Emergency Medicine, Los Angeles, California, USA; 7 Division of Emergency Medicine, Department of Pediatrics, Duke University School of Medicine, Durham, North Carolina, USA; 8 Department of Emergency Medicine, Harbor-University of California, Los Angeles (UCLA) Department of Emergency Medicine, Torrance, California, USA; 9 Department of Emergency Medicine, Prisma Health, Greenville, South Carolina, USA; 10 Department of Emergency Medicine, Weill Cornell Medicine, New York, New York, USA; 11 Department of Pediatrics, Dell Medical School, The University of Texas at Austin, Austin, Texas, USA; 12 Department of Pediatric Emergency Medicine, Nemours Children’s Hospital, Orlando, Florida, USA; 13 Department of Pediatrics and Emergency Medicine, Emory University School of Medicine, Atlanta, Georgia, USA; 14 Department of Emergency Medicine, Lincoln Medical Center, Bronx, New York, USA

**Keywords:** adolescent substance use, emergency department screening, SBRIT, brief intervention, linkage to care

## Abstract

Adolescent substance use remains a major public health concern, and the emergency department (ED) represents a critical point of contact for identification and early intervention. Many adolescents rely on the ED as their primary or sole source of health care, making missed opportunities for screening particularly consequential. Although overall substance use rates among youth have stabilized or declined in recent years, drug-related mortality, especially from illicitly manufactured fentanyl, has risen sharply, highlighting the need for proactive detection and intervention.

Professional organizations recommend universal, validated screening for substance use in adolescents, yet implementation in ED settings remains inconsistent due to workflow constraints, confidentiality concerns, and limited provider training. Validated tools, such as the Car, Relax, Alone, Forget, Friends, Trouble (CRAFFT) questionnaire, the screening to brief intervention (S2BI) tool, and the brief screener for tobacco, alcohol, and other drugs (BSTAD) tool, allow rapid, developmentally appropriate screening. They can be integrated into electronic health records (EHRs) or self-administered digital formats. Evidence suggests that electronic or kiosk-based screening increases disclosure and detection without adversely affecting throughput.

Screening alone is insufficient; pairing detection with brief motivational interventions and facilitated referral to treatment improves short-term outcomes and linkage to care. However, follow-up rates remain suboptimal, underscoring the need for structured referral pathways, behavioral health integration, and technology-enabled supports such as text-based reminders or telehealth. Universal, developmentally tailored screening in the ED, combined with brief intervention and active linkage to outpatient care, offers a practical strategy to reduce substance-related morbidity and improve long-term outcomes for adolescents.

## 1 Introduction

The emergency department (ED) is an important point of contact for detecting substance use in adolescents. Substance use-related ED visits among youth increased 47.9% from 2016 to 2021 at US children's hospitals, rising from 2.8% to 3.4% of all youth ED visits between 2018 and 2023.^1^^,^^2^ Adolescents differ from adults in 3 critical respects: (1) ongoing brain maturation heightens vulnerability to substance-induced cognitive and emotional harm; (2) confidentiality and minor consent laws vary by state and influence what can be asked, disclosed, and treated; and (3) earlier identification affords a wider window to avert long-term consequences and development of substance use disorders (SUDs).^3^

Recognizing these distinctions, the American Academy of Pediatrics (AAP) recommends universal validated screening of all adolescents at every well visit.^4^ The Substance Abuse and Mental Health Services Administration (SAMHSA) recommends screening, brief intervention, and referral to treatment (SBIRT) as part of routine health care.^5^^,^^6^ The American College of Surgeons (ACS) requires universal screening of admitted patients with trauma older than 12 years for alcohol misuse.^7^ Despite these recommendations, implementation remains limited. A recent Centers for Disease Control and Prevention (CDC) survey found that only 56.9% of clinicians who treat adolescents reported screening for SUDs at every well visit, and only 30.7% routinely offered all components of SBIRT.^8^

Self-reported substance use among US adolescents has declined substantially since the COVID-19 pandemic onset.^9^ In 2025, past 12-month cannabis use was 26% among 12th graders and 16% among 10th graders, the lowest levels in three decades. Alcohol, cannabis, and nicotine vaping remain the most commonly used substances, with no post-pandemic rebound observed.^10^

Despite these declines in self-reported use, drug-related mortality is rising far more sharply in adolescents than in adults. Overdose deaths among persons aged 14 to 18 years increased 94% from 2019 to 2020, with approximately 90% involving opioids and 83.9% involving illicitly manufactured fentanyl (IMFs); notably, only 35% of decedents had documented prior opioid use history.^11^ Mortality increases have been steepest in Black and American Indian/Alaska Native adolescents. These inequities highlight the need for ED screening protocols that are culturally responsive and trauma-informed.^3^^,^^12^

## 2 Clinical Presentation

Adolescent substance use often presents with heterogeneous features, including mood and behavioral changes, impaired cognition, and altered mental status.^13^ Central nervous system impairments are common; 1 study found 81% of adolescents presenting with drug misuse were symptomatic, most frequently with psychosis, agitation, and coma.^13^ Cannabinoid hyperemesis syndrome (CHS), once rare, is now a frequent clue to heavy cannabis or high-potency vaping product use; CHS presents with cyclic vomiting, nausea, and abdominal pain and is rising in prevalence.^14^^,^^15^ Compared with adults, teens more commonly combine cannabis and nicotine ("cross-fading") and may conceal use, resulting in atypical presentations such as cyclic vomiting with normal vital signs. Adolescents with chronic use may develop withdrawal during observation: opioid withdrawal manifests as agitation, gastrointestinal distress, and piloerection or benzodiazepine withdrawal presenting with tremor, hallucinations, and seizures. Unlike adult patients, where long histories guide prediction, teen withdrawal is often unanticipated because patients and caregivers underreport duration and dose or may not recognize the degree of dependence. Rapid bedside screening enables preemptive management and avoids misattribution to anxiety or infection.

Because brain development continues into the mid-20s, early substance exposure accelerates structural and functional changes linked to a higher risk of later SUDs.^16^ Adolescents with chronic medical conditions had 8- to 10-fold higher odds of substance-related ED visits during the COVID-19 era, a vulnerable subgroup that routine screening seldom captures.^17^ Critically, approximately 20.9% of adolescent substance abuse ED visits include a co-occurring mental health diagnosis, mandating dual assessment of suicidality and substance use as standard practice.^18^

## 3 Screening Tools and Implementation

The use of validated screening tools significantly improves the identification of substance use and increases the likelihood of early intervention.^19^^,^^20^ Brief screening tools using past-year frequency questions, the screening to brief intervention [S2BI] and the brief screener for tobacco, alcohol, and other drugs [BSTAD] screening tools demonstrate high agreement with Diagnostic and Statistical Manual of Mental Disorders, Fifth Edition (DSM-5) diagnostic criteria (area under the curve value ranges from 0.89 to 1.0).^20^ Adolescents report greater willingness to disclose substance use via electronic self-administration, with 90% reporting higher comfort and honesty compared to paper-based methods.^21^ The Car, Relax, Alone, Forget, Friends, Trouble (CRAFFT) questionnaire; the S2BI screening tool; the BSTAD; the alcohol use disorders identification test (AUDIT); and the drug abuse screening test (DAST) screening tools are among the tools suitable for ED use.^22^^,^^23^ The AAP specifically recommends the CRAFFT questionnaire, the S2BI, and the BSTAD screening tools for adolescent substance use screening.^23^ Their characteristics are compared in Table 1.^20^^,^^24^, ^25^, ^26^, ^27^, ^28^, ^29^, ^30^

**Table 1:** Validated adolescent screening tools for the emergency department (ED).^20^^,^^24^, ^25^, ^26^, ^27^, ^28^, ^29^, ^30^

Tool	Time	Substances	Scoring	Sn/Sp	ED suitability
CRAFFT questionnaire 2.1 + N^24^^,^^25^	2 min	Alcohol, cannabis, other drugs, and nicotine	Score 0 = low risk; scores 1-2 = moderate risk; and score ≥ 3 = high risk (≥79% likelihood of SUD)	Sn, 0.73-0.94 Sp, 0.64-0.96 varies by cut-off and substance	Best-studied in PED; electronic self-administration validated; EHR-integrable.
S2BI screening^20^^,^^26^	20 s	Tobacco, alcohol, cannabis, prescription drugs, and other drugs	Frequency-based risk levels: none, once/twice, monthly, weekly+	Sn, 0.90-1.00 Sp, 0.85-0.97 for past-year use	Ultrabrief; ideal as prescreen in a 2-step algorithm; electronic format available
BSTAD screening^20^^,^^27^	1-2 min	Tobacco, alcohol, cannabis, prescription drugs, and other drugs	Frequency-based; identifies past-year use with substance-specific thresholds	Sn, 0.84-0.95 Sp, 0.83-0.97	Validated for aged 12-17 years; suitable for self- or clinician-administration
AUDIT test^28^^,^^29^	2-3 min	Alcohol only	10-item, 0-40 scale; score ≥ 8 = hazardous use; and score ≥ 20 = likely dependence	Sn, 0.80-0.97 Sp, 0.78-0.95 in adult populations; limited adolescent data	Primarily validated in adults; AUDIT-C test (3-item) is useful as a rapid alcohol prescreen
DAST-10 test^30^	2-3 min	All nonalcohol drugs	10-item, yes/no; score, 1-2 = low; score 3-5 = moderate; and score ≥ 6 = substantial/severe	Sn, 0.80-0.95 Sp, 0.71-0.94 Adult-derived; limited pediatric data	Complements AUDIT test for nonalcohol drugs; less commonly used alone in PEDs

AUDIT, alcohol use disorders identification test; AUDIT-C, alcohol use disorders identification test-concise; BSTAD, brief screener for tobacco, alcohol, and other drugs; CRAFFT, car, relax, alone, forget, friends, trouble; DAST-10, drug abuse screening test-10; EHR, electronic health record; IVDU, intravenous drug use; PED, pediatric emergency department; S2BI, screening to brief intervention; Sn, Sensitivity; Sp, Specificity; SUD, substance use disorder.

Performance metrics reflect published validation studies; values vary by population, cut-off, and substance.

## 4 Implementation Strategies

Embedding the screen in triage or first nurse assessment creates a "hard stop" requiring completion or documented refusal. Across 7 pediatric trauma centers, SBIRT adoption increased from 11% to 73% after implementation of institutional policies.^31^ Kiosk-based self-administration significantly increases disclosure of high-risk substance use; in 1 ED study, patients disclosed hazardous drinking and high-risk drug use at significantly higher rates via kiosk (odds ratio of 22.3 for drug use; *P* < .0001).^32^^,^^33^ A 2-step algorithm preserves speed: the S2BI screening tool as a prescreen, with the full CRAFFT questionnaire administered only if any use is endorsed.^26^ The ideal workflow treats substance use screening like a vital sign: the nurse initiates, the EHR auto-scores, and a social worker or peer coach delivers the brief intervention and schedules follow-up.

Despite the demonstrated effectiveness of screening tools, several barriers hinder routine use. Reported barriers include time constraints, limited resources, lack of training, and scarce referral options.^33^ A national cross-setting evaluation of more than 1266 sites that work with youth found implementation routinely hampered by workflow time, reimbursement, and scarce referral options, challenges mirrored in emergency care.^34^ Clinician readiness is uneven: only 20.9% of ED clinicians indicated readiness to initiate buprenorphine, and buprenorphine dispensing to youth remains low, with pediatricians accounting for less than 2% of prescriptions dispensed to adolescents.^35^, ^36^, ^37^ Surveys indicate that lack of training and protocols remain primary barriers.^38^, ^39^, ^40^ Linguistic and cultural validity also matter: straight translation of CRAFFT questionnaire items can miss colloquial drug slang, and several centers are piloting multilingual versions before broader rollout.^41^

## 5 Structured Postscreening Workflow

Upon completion of screening, the result should be immediately risk-stratified using the embedded scoring algorithm. For the CRAFFT questionnaire, a score of 0 indicates low risk, 1 to 2 suggests moderate risk, and a score of 2 or greater is generally considered a positive screen warranting further assessment.^4^^,^^42^ Beinenson et al^43^ demonstrated that CRAFFT questionnaire scores of 3 or greater, associated with a 79% or greater likelihood of meeting SUD criteria, triggered referral to addiction treatment. In contrast, lower scores prompted general outpatient psychiatric evaluation.

For adolescents with signs of acute intoxication, withdrawal, suicidal ideation, or other medical or psychiatric emergencies, immediate stabilization and safety assessment take precedence over brief intervention.^4^^,^^44^ Dual assessment for suicidality and substance use is recommended, given that approximately 1 in 5 (20.9%) adolescent substance-related ED visits include a co-occurring mental health diagnosis.^18^ The AAP recommends screening all youth aged 12 years and older for suicide risk at least annually, and the Ask Suicide-Screening Questions (ASQ) toolkit brief suicide safety assessment includes evaluation of current symptoms, including substance use.^45^ The The American Academy of Child and Adolescent Psychiatry (AACAP)'s 2025 clinical practice guideline reinforces the need for concurrent evaluation and suggests safe and effective short-term treatments, including brief motivational interviewing (MI) for alcohol use, family therapy or cognitive-behavioral therapy (CBT) for alcohol use disorder with or without other drug use; MI plus CBT for illicit drug disorders; and longer-term buprenorphine treatment for opioid use disorder (OUD).^46^

Once the patient is medically stable, the clinician proceeds through a stepwise pathway: risk stratification, brief MI, substance-specific management, harm reduction, and facilitated referral to ongoing care (Table 2). The EHR should automate scoring, prompt the next steps based on risk level, and document all interventions and referrals in a confidential section of the chart. Monthly dashboards tracking screening rate, brief intervention completion, and referral attendance help sustain adherence and highlight equity gaps.

**Table 2:** Postpositive screen decision pathway.

Risk level	Screen result	Recommended ED actions	Disposition/referral
Immediate safety concern (any screen result)	Acute intoxication, withdrawal, suicidal ideation, or psychiatric emergency	1. Medical stabilization 2. Suicide risk assessment (concurrent with SU evaluation) 3. Initiate pharmacotherapy as indicated (eg, buprenorphine for OUD withdrawal) 4. Proceed to BI once stable	Inpatient admission, psychiatric evaluation, or crisis stabilization as clinically indicated
High risk	CRAFFT questionnaire score ≥3 S2BI screening: weekly+ use Any opioid/injection drug use	1. Brief MI (5-15 min) 2. Substance-specific intervention (see Table 4) 3. Harm reduction: naloxone, fentanyl test strips, overdose education 4. Warm handoff to behavioral health or peer recovery coach 5. Schedule an outpatient appointment before discharge	Referral to addiction medicine or specialized adolescent SUD treatment; outpatient appointment within 7 d; automated text follow-up
Moderate risk	CRAFFT questionnaire scores 1-2 S2BI screening: monthly use	1. Brief MI (5-10 min) 2. Psychoeducation and harm reduction counseling 3. Assess for co-occurring mental health concerns	Referral to outpatient behavioral health or general psychiatric evaluation; notify PCP with patient consent
Low risk	CRAFFT questionnaire score 0 S2BI screeningt: no use/once or twice	1. Positive reinforcement 2. Anticipatory guidance on substance use risks	Routine follow-up with PCP; no specialty referral required

BI, brief intervention; CRAFFT, car, relax, alone, forget, friends, trouble; ED, emergency department; MI, motivational interviewing; OUD, opioid use disorder; PCP, primary care provider; S2BI, screening to brief intervention; Su, substance use; SUD, substance use disorder.

## 6 Brief MI

Brief MI is the universal first-line intervention recommended by the AAP, AACAP, and the National Institute on Alcohol Abuse and Alcoholism (NIAAA) for all adolescents with a positive substance use screen.^4^^,^^46^^,^^47^ MI is a collaborative, nonjudgmental counseling style aimed at enhancing intrinsic motivation for change. A meta-analysis of 22 randomized controlled trials found that MI reduces heavy alcohol use days by 0.7 days per month (95% credible interval [CrI], −1.6 to 0.02), alcohol use days by 1.1 days per month (95% CrI, −2.2 to −0.3), and overall substance-related problems by a standardized net mean difference of 0.5 (95% CrI, −1.0 to 0), though it did not significantly reduce cannabis use days (net mean difference −0.05 days per month; 95% CrI, −0.26 to 0.14).^48^ Even a single session of 5 to 15 minutes can increase readiness to change.^46^^,^^47^

A practical MI dialogue for the ED setting is outlined in Table 3, adapted from the NIAAA Practitioner's Guide.^47^ MI can be delivered by a range of ED staff, including social workers, nurses, or peer recovery coaches, following brief training. Fidelity to MI principles is associated with greater effectiveness.^49^ Structured EHR templates can support consistent delivery in busy settings. Gaume et al^50^ demonstrated in a randomized clinical trial that a structured brief MI in the ED for alcohol-intoxicated young adults, incorporating booster sessions, prevented escalation of heavy drinking days compared to brief advice alone.^50^

**Table 3:** Sample brief motivational interviewing (MI) dialogue for the emergency department (ED).

MI Step	Example clinician language
1. Ask permission	“Your screening showed some substance use. Would it be okay if we talked about that for a few minutes?”
2. Express empathy/explore context	“Tell me a little about what’s been going on. What does your use typically look like?”
3. Develop discrepancy	“You mentioned wanting to make the varsity team. How does the drinking fit with that goal?”
4. Elicit change talk	“What concerns you most about your use? What would be different if you cut back?”
5. Roll with resistance/affirm autonomy	“It’s completely your decision. I’m not here to tell you what to do—just to make sure you have the information you need.”
6. Provide feedback with permission	“Would it be alright if I shared what we know about how [substance] affects the developing brain? At your age, the brain is still building connections for decision-making, and regular use can slow that down.”
7. Support self-efficacy	“The fact that you’re thinking about this tells me you take your health seriously. That’s a real strength.”
8. Collaborative goal-setting	“What feels like a realistic next step you’d be willing to try? Even a small change counts.”
9. Facilitate referral/summarize	“Let me make sure I heard you right: [summarize]. I’d like to connect you with someone who can help with the next steps—would that be okay?” Total time: 5-15 min. Can be delivered by a physician, social worker, nurse, or peer recovery coach.

## 7 Substance-Specific Management

Each substance class warrants targeted management in the ED (Table 4). Opioid use, particularly involving illicitly manufactured fentanyl, carries the highest risk of overdose death; 90% of adolescent overdose deaths now involve fentanyl.^51^ Any adolescent screening positive for opioid use should be assessed for OUD and withdrawal, with buprenorphine initiation offered when OUD is identified.^46^^,^^51^^,^^52^ The standard induction protocol begins with assessment using the clinical opioid withdrawal scale (COWS) score, with initiation when the score reaches 8 or higher. For short-acting opioids, this typically corresponds to 6 to 12 hours since last use; for fentanyl, at least 12 to 24 hours may be required due to prolonged tissue retention and elevated risk of precipitated withdrawal.^51^ Sublingual buprenorphine/naloxone dosing begins at 2 to 4 mg with reassessment and titration as needed (see Table 4 for full dosing protocol).^51^ A recent multicenter randomized trial (ED INNOVATION [Emergency Department-INitiated bupreNOrphine and VAlidaTIOn Network]) demonstrated that a single 24 mg subcutaneous extended-release buprenorphine injection can be safely administered in the ED, even in patients with minimal to mild withdrawal, with a precipitated withdrawal rate of less than 1% across 2000 initiations.^53^ Postdose monitoring for 1 to 2 hours with serial COWS score assessments is essential; adjunctive medications may be used for symptomatic relief.^53^^,^^54^ Importantly, the regulatory barrier posed by the federal DATA-2000 "X-waiver" has been removed; under Section 1262 of the 2023 Consolidated Appropriations Act (MAT Act), any clinician with a standard Drug Enforcement Administration (DEA) registration with Schedule III authority may prescribe buprenorphine for OUD without a special waiver.^53^

**Table 4:** Substance-specific ED intervention quick reference.

Substance	ED intervention	Pharmacotherapy	Referral pathway
Opioids	Assess for OUD and withdrawal (COWS) Initiate buprenorphine if COWS score ≥ 8 Naloxone distribution and overdose education Fentanyl test strip provision If IVDU: HIV/HCV screen, syringe exchange referral	Buprenorphine/naloxone: start 2-4 mg SL; reassess 30-60 min Additional 2-4 mg PRN; up to 8-12 mg d 1 Up to 16 mg for fentanyl exposure Extended-release: 24 mg SQ (single dose, < 1% PW rate) Discharge Rx: 8-16 mg/d bridge Contraindications: hypersensitivity, severe hepatic impairment, acute sedative intoxication, and recent methadone use	Outpatient follow-up within 7 d Addiction medicine or adolescent SUD specialist naloxone Rx at discharge
Alcohol	Brief MI (first-line) Assess for AUD, withdrawal risk Medical stabilization if intoxicated	No US FDA-approved pharmacotherapy for adolescent AUD Manage acute withdrawal per clinical judgment	CBT or family-based therapy Schedule first appointment before discharge
Cannabis	Brief MI Cannabinoid hyperemesis education if applicable Assess frequency and functional impact	No US FDA-approved pharmacotherapy Contingency management as adjunct (evidence-based for cannabis)	MI + CBT for cannabis use disorder Integrated behavioral health
Nicotine/vaping	Brief MI and psychoeducation Assess the severity of tobacco use disorder	NRT counseling for moderate/severe dependence No bupropion/varenicline data in <18 y	Tobacco cessation program PCP follow-up
Polysubstance	MI is addressing all substances concurrently Prioritize highest-acuity substance (eg, opioids > others) Comprehensive harm reduction	Per individual substance Contingency management as adjunct (stimulant/cannabis)	Integrated behavioral health care Adolescent SUD specialist Consider residential if severe/unstable

AUD, alcohol use disorder; CBT, cognitive behavioral therapy; COWS, clinical opioid withdrawal scale; ED, emergency department; HCV, hepatitis C virus; IVDU, intravenous drug use; MI, motivational interviewing; NRT, nicotine replacement therapy; OUD, opioid use disorder; PRN, as needed; PW, precipitated withdrawal; Rx, therapy; SL, sublingual; SQ, subcutaneous; SUD, substance use disorder; US FDA, United Stataes Food and Drug Administration.

Naloxone distribution is recommended for all adolescents at risk for opioid overdose, including those using stimulants from nonmedical sources, given widespread fentanyl contamination.^55^ Naloxone is now approved for over-the-counter sales to individuals younger than 18 years, yet dispensing rates remain far below need, with only 50.9 prescriptions per 100,000 adolescents in 2022, a 669% increase from 2017, but still insufficient.^56^ Fentanyl test strips have high sensitivity (>95%) for detecting fentanyl, though they may not detect all analogs such as carfentanil and should be offered with education on their use and limitations.^57^ For adolescents who inject drugs, infectious disease screening for HIV and hepatitis C infections, safe injection education, and referral to syringe exchange programs are recommended.^58^ For alcohol, cannabis, nicotine, and polysubstance use, brief MI is the first-line ED approach, with substance-specific referral pathways.^46^^,^^48^ For stimulant use disorder, contingency management in combination with other behavioral interventions has demonstrated efficacy; for cannabis use disorder, CBT and motivational enhancement therapy are suggested first-line treatments.^55^^,^^59^^,^^60^

## 7 Linkage to Care, Cost, and Implementation

The persistent gap between ED intervention and treatment uptake is a central challenge. Studies show highly variable linkage rates: without active intervention, only 15.9% of patients engage in outpatient treatment within 30 days, while substance use navigator programs achieve 50.4% engagement. Adolescents rarely self-refer to treatment.^61^ Active facilitation strategies are essential. Warm handoff models have yielded substantially higher referral attendance rates than passive discharge advice.^62^^,^^63^ Scheduling the first outpatient appointment during the ED stay and providing transportation vouchers or telehealth options addresses common adolescent barriers such as reliance on caregivers and limited independence. Text messaging interventions reduce substance use behaviors in adolescents and may improve treatment engagement.^64^

Peer recovery coaches are effective for adolescents, leveraging shared lived experience to build rapport and facilitate system navigation.^65^, ^66^, ^67^ In rural EDs, telehealth can connect adolescents with peer coaches and addiction specialists remotely. For OUD, initiating buprenorphine during the ED visit is associated with 5- to 6-fold higher odds of treatment engagement at 30 days compared to referral alone, yet more than 80% of pediatric emergency medicine providers have never started buprenorphine in an adolescent.^38^^,^^68^^,^^69^

Cost and resource concerns are frequently cited as barriers to implementation. The screening itself adds minimal time: a 2-step algorithm (S2BI prescreen followed by full CRAFFT questionnaire only if use is endorsed) keeps added triage time under 1 minute, and delegation to nursing or digital self-administration reduces physician burden.^26^^,^^32^ The cost-effectiveness of SBIRT in adults is well established, with a 21% reduction in total health care costs and significant decreases in repeat ED visits over 1 year.^70^ In adolescents, direct cost-effectiveness data are limited, though a multisite initiative screening over 140,000 youth demonstrated feasibility and scalability.^34^ SBIRT sustainability is supported by insurance billing codes (Current Procedural Terminology [CPT] 99408/99409 for screening and brief intervention; the Healthcare Common Procedure Coding System [HCPCS] H0049/H0050 for alcohol and drug screening), though grant funding remains common.^34^

Implementation facilitators include standing orders and protocols for immediate intervention, EHR-integrated decision support and automated workflows, clear delineation of staff roles, dashboard metrics with audit-and-feedback mechanisms, and robust community partnerships.^71^, ^72^, ^73^ The most commonly cited barriers are lack of time, knowledge, and resources.^74^ EHR-based algorithms and artificial intelligence (AI) tools can flag at-risk adolescents, automate resource referrals, and prompt linkage to services, reducing documentation burden. Natural language processing (NLP) has identified opioid use disorders from unstructured EHR notes with high accuracy (area under the receiver operating characteristic curve value, 0.88-0.97) and may enable real-time risk stratification.^75^^,^^76^

## 8 Confidentiality and Equity

Confidentiality is foundational to adolescent care and directly influences willingness to disclose substance use. The Health Insurance Portability and Accountability Act (HIPAA) Privacy Rule establishes baseline protections, and Title 42 of the Code of Federal Regulations (CFR) Part 2 provides more stringent protections for SUD-related records, prohibiting disclosure, including to parents, without the minor's written consent.^77^ State laws vary regarding the age at which minors can consent to SUD treatment and the circumstances requiring parental notification; clinicians must explain the limits of privacy and negotiate family involvement.^77^ The AAP recommends that confidential care be documented in a sequestered portion of the EHR unavailable to parents and that billing mechanisms avoid inadvertent disclosure through insurance statements.^23^^,^^78^ Confidentiality may be breached when escalating patterns of use, high-risk behaviors such as driving while intoxicated, or use of substances with high mortality risk (eg, fentanyl) warrant disclosure to ensure the adolescent's safety.^78^

Equity-focused strategies are essential to ensure that post-screening interventions address disparities affecting racial and ethnic minorities; lesbian, gay, bisexual, transgender, and queer/questioning (LGBTQ+) youth; and youth with chronic illness. Significant racial and ethnic disparities exist in SUD treatment: Black and Latinx youth experience lower retention in SUD treatment compared to White patients, and Black patients are 70% less likely to receive a prescription for buprenorphine than White patients when controlling for payment method, sex, and age.^79^ The AAP recommends culturally and linguistically adapted screening and interventions, trauma-informed care, and social determinants of health (SDOH) screening with active resource linkage.^16^ For LGBTQ+ youth, strategies include using chosen names and pronouns, employing gender-inclusive language, and providing privacy in triage.^80^ A 2025 meta-analysis found that sexual minority youth report significantly higher rates of substance use than heterosexual peers, with the greatest disparities among plurisexual youth, sexual minority girls, and younger adolescents.^81^

## 9 Conclusions

Universal, validated electronic screening can be completed in minutes, detect hidden substance use, and close longstanding equity gaps in identification. When paired with a structured postscreening workflow, brief motivational intervention, substance-specific management, harm reduction, and active facilitation of referral, screening translates into higher linkage-to-care rates and measurable short-term reductions in alcohol- and cannabis-related harms. Initiation of buprenorphine in the ED, now standard for adults and feasible for youth, should be supported by standing protocols in every pediatric emergency setting. The AACAP's 2025 clinical guideline recommendations and removal of the X-waiver requirement allow for expanded use of pharmacotherapy for adolescents with SUD in the ED. Future research should prioritize adolescent-specific cost-effectiveness analyses, longitudinal outcome studies beyond 12 months, validation of screening tools for emerging substances, and integration of AI and NLP tools for real-time risk stratification and care coordination.

## Author Contributions

SS contributed original writing for section content and formatted, collated, and edited the final draft of the manuscript. SA, IB, KB, K. Brown, TC, CC, IC, AMD, SL, TR, MS, CS, and MW contributed to writing sections of content.

## Funding and Support

By JACEP Open policy, all authors are required to disclose any and all commercial, financial, and other relationships in any way related to the subject of this article as per ICMJE conflict of interest guidelines (see www.icmje.org). The authors have stated that no such relationships exist.

## Conflict of Interest

All authors have affirmed they have no conflicts of interest to declare.
